# A frugal implementation of Surface Enhanced Raman Scattering for sensing Zn^2+^ in freshwaters – In depth investigation of the analytical performances

**DOI:** 10.1038/s41598-020-58647-7

**Published:** 2020-02-05

**Authors:** Gwennhaël Brackx, Damien Guinoiseau, Ludovic Duponchel, Alexandre Gélabert, Victoria Reichel, Samia Zrig, Jean-Marc Di Meglio, Marc F. Benedetti, Jérôme Gaillardet, Gaëlle Charron

**Affiliations:** 1 Laboratoire Matière et Systèmes Complexes, UMR 7057, Université Paris Diderot, Sorbonne Paris Cité, CNRS, 10 rue Alice Domon et Léonie Duquet, 75205 Paris, cedex 13 France; 2 Institut de Physique du Globe de Paris, Sorbonne Paris Cité, CNRS UMR 7154, 1 rue Jussieu, 75005 Paris, France; 3 LASIR CNRS UMR 8516, Université de Lille, Sciences et Technologies, 59655 Villeneuve d’Ascq Cedex, France; 4 ITODYS, UMR 7086, Université Paris Diderot, Sorbonne Paris Cité, CNRS, 15 rue J-A de Baïf, 75205 Paris, cedex 13 France

**Keywords:** Environmental monitoring, Sensors

## Abstract

Surface Enhanced Raman Scattering (SERS) has been widely praised for its extreme sensitivity but has not so far been put to use in routine analytical applications, with the accessible scale of measurements a limiting factor. We report here on a frugal implementation of SERS dedicated to the quantitative detection of Zn^2+^ in water, Zn being an element that can serve as an indicator of contamination by heavy metals in aquatic bodies. The method consists in randomly aggregating simple silver colloids in the analyte solution in the presence of a complexometric indicator of Zn^2+^, recording the SERS spectrum with a portable Raman spectrometer and analysing the data using multivariate calibration models. The frugality of the sensing procedure enables us to acquire a dataset much larger than conventionally done in the field of SERS, which in turn allows for an in-depth statistical analysis of the analytical performances that matter to end-users. In pure water, the proposed sensor is sensitive and accurate in the 160–2230 nM range, with a trueness of 96% and a precision of 4%. Although its limit of detection is one order of magnitude higher than those of golden standard techniques for quantifying metals, its sensitivity range matches Zn levels that are relevant to the health of aquatic bodies. Moreover, its frugality positions it as an interesting alternative to monitor water quality. Critically, the combination of the simple procedure for sample preparation, abundant SERS material and affordable portable instrument paves the way for a realistic deployment to the water site, with each Zn reading three to five times cheaper than through conventional techniques. It could therefore complement current monitoring methods in a bid to solve the pressing needs for large scale water quality data.

## Introduction

The management of water resources necessitates large volumes of water quality data so as to identify sources of pollution, assess contaminant levels in aquatic ecosystems, control the safety of drinking water, safeguard the reuse of wastewater or ascertain the efficiency of remediation actions^1^. This need was recognised by the European Union and the United States as early as in the mid-nineties, in reports where representatives of both governments recommended moving away from laboratory-based analytical procedures, which could not sustain suitable throughputs of water quality data^2,3^. They called instead for the development of analytical methods and sensors operated directly on site, at a high temporal frequency and possibly in a continuous fashion. Yet in the current decade, major international (development) organisations are still calling for improved analytical tools capable of high throughput water quality monitoring^4–6^.

With current monitoring methods, large-scale water quality data comes at large-scale budget. For instance, heavy metal monitoring costs on the order of 25 to 50 € per analysis in a public or private sector analytical laboratory using current golden standard techniques (Atomic Absorption Spectroscopy (AAS), Inductively Coupled Plasma Optical Emission Spectroscopy (ICP-OES) or Inductively Coupled Plasma Mass Spectrometry (ICP-MS))^7,8^. These prices per analysis reflect the cost of the instrument (50 k€ for an entry-level ICP-OES), the use of hazardous and expensive consumables to run the measurements (argon gas cylinders in the case of ICP-OES/MS, ultrapure concentrated acids) and the relative complexity of the measurement procedure (such as acid digestion). In addition, these techniques are fairly impractical outside of a laboratory environment, and therefore require the samples to be shipped to the analytical facility, which results in delays in reading the outcome and can be logistically challenging in remote or unsafe areas.

Surface Enhanced Raman Scattering (SERS) is a spectroscopic technique that holds potential to deliver contaminant analysis directly on site, for cheaper. SERS is the phenomenon by which an analyte sees its Raman spectrum enhanced by several orders of magnitude when sitting in a so-called “hot spot”, namely in the close vicinity of a nanostructured plasmonic materials displaying sharp edges, tips or gaps^9^. The technique is said to be extremely sensitive: dedicated articles routinely report limits of detection (LODs) in the µM to the fM range^10–14^, and down to single molecules under certain conditions^15^. Moreover, SERS spectra usually encompass a rich collection of peaks with narrow linewidth. The SERS spectrum of an analyte can thus be viewed as its specific fingerprint: it cannot be mistaken for that of another species, which allows for detection in complex samples. Finally, SERS can be performed using affordable and handy portable Raman spectrometers costing on the order of 8–20 k€.

SERS has been the scene of important research developments in the fields of biosensing^16^, food analysis^17–21^, and environmental sensing^22,23^. However, and despite having been highlighted as a promising technique for field analysis twenty years ago^3^, real world applications of SERS are still non-existent, there are no commercial SERS detection kits, and the technique is mostly unknown to end-users such as geoscientists or biologists beside the occasional scientific curiosity. We believe the reasons for this arrested development are two-fold.

First, analytical implementations of SERS have generally overlooked the issue of the accessible scale of measurements, which is generally controlled by the time and cost-efficiency of two distinct steps of the overall analytical procedure: the synthesis of the SERS-active material and the preparation of the sample. Often, the plasmonic material used is produced at a small scale because it is synthetically challenging and/or time-consuming to produce, when the synthetic scheme involves multiple steps for instance. Often too, the sample preparation time is highly limiting, with reported times on the order of hours being common, as is the case when the sample needs to be left to dry on a wafer (a situation that is likely to be impractical outside of the laboratory). As a result, the corresponding number of measurements presented in reports rarely exceed 30.

Second, and mostly because of this scarcity of measurements, the analytical figures of merits that matter most to the end-user are very often omitted or improperly reported. Generally, the LOD is used as the only metric to evaluate the performances of the SERS sensing scheme. Yet for routine application, accuracy in a specific range of concentrations covering those actually encountered in samples is a more useful indicator. Both LOD and accuracy require significant statistical samples to be derived. The usually low availability of the SERS sensing material makes it difficult to perform such rigorous, statistically meaningful evaluation of the analytical performances of the SERS detection.

In this study, we attempt an implementation of SERS detection in the case of the heavy metal contaminant Zn^2+^ rooted in the principle of frugality, namely in the aspiration of providing just what is needed, at no unnecessary cost. This consideration is often at the heart of programs fostering technological transfers to developing countries, which are the most in need of affordable tools to monitor water quality^24^. Importantly, we investigate the resulting performances of the sensor in depth, using the metrics most likely to inform the choice of potential end-users.

Zn^2+^ was chosen as the target analyte as it is both a contaminant in itself (considered a priority by some EU member states) and an indicator of other heavy metal contaminants. Zinc is an essential nutrient to plants and animals. Both deficiencies and overload are considered harmful, leading for instance to impaired plant growth^25^, or adverse neurological effects in humans^26^. Zn^2+^ discharges from mining and smelting plants often go hand in hand with contaminations by ultra-trace elements Cd, Pb and Hg^8,27–29^. These three elements are far more concerning to the health of ecosystems or human populations but being 1000 to 10000 times less abundant than Zn, they are also a lot more difficult to monitor. Zn^2+^ concentration can therefore be used as a proxy for contamination by heavier and more toxic metals, when a direct assessment cannot be conducted due to cost or time constraints.

The proposed sensing method exploits a SERS active material that can be synthesised in one step and on a large scale, from chemical components that can be easily sourced, a simple mixing procedure for the preparation of the analytical sample that could be reproduced by a non-chemist and a portable Raman spectrometer that could eventually be deployed on field. Using this frugal set-up, we acquire a dataset much larger than what is conventionally done in the field of SERS. From it, we estimate analytical figures of merit that are critical to the end-user, namely sensitivity range, trueness, precision, and give a tutorial-like methodological framework to derive those figures, inspired by recent recommendations on good analytical practices in the field of SERS detection^30^. Moreover, we compare the consolidated cost of our SERS readings to those of golden standard methods of Zn quantification.

Ultimately, we demonstrate that our frugal set-up is capable of accurately sensing Zn^2+^ at environmentally relevant concentrations, despite displaying a sensitivity much weaker than commonly reported for SERS sensors. Critically, we find that it can do so on scales suitable to run thousands of measurements, using an instrument that can be brought directly on site, at a financial cost 3 to 5 times smaller than current golden standard methods of heavy metal determination.

## Results

### Chemical sensing scheme

As an atomic ion, Zn^2+^ cannot display a SERS signal on its own. One strategy to impart an atomic ion with a SERS signal consists in having it interact with a Raman-active chelator which will see its own SERS spectrum modified upon recognition of the analyte^31^. Zn^2+^, Hg^2+^, Co^2+^, Cu^2+^ and Cl^−^ have been quantified in this way using conventional metal cation and anion ligands^32–35^. However, these studies exploited schemes that were not suitable for large measurement campaigns because of the high manufacturing cost of the SERS-active plasmonic nanomaterials or the time-consuming procedures for sample preparation. Here, we reinvest this strategy in a detection scheme in which the chemical components can be sourced on a scale worth 5000 measurements and where sample preparation and spectral acquisition are completed in four minutes.

**Figure 1:**
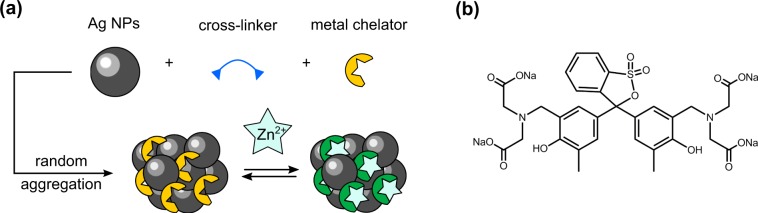
Chemical sensing scheme of Zn^2+^ ions (**a**) and structure of the metal chelator Xylenol Orange (**b**).

Briefly, silver nanoparticles (Ag NPs) are aggregated in the presence of a selective ligand of Zn^2+^ through addition of a cross-linking agent (Fig. 1). The aggregates are then exposed to the analyte aqueous solution, upon which the SERS spectrum of the trapped receptors is modified as they bind Zn^2+^ ions.

Citrate-stabilised Ag NPs synthesised by the Lee-Meisel route were selected for their ease of synthesis and because they notoriously give rise to intense SERS spectra upon aggregation, albeit with irreproducible count numbers. Spermine was chosen as the cross-linking agent. This 200 g.mol^−1^ molecule bears four amines that can bridge Ag NPs through a mix of coordination and electrostatic interactions (Fig. S1). The ratio of spermine to Ag NPs was adjusted so that, through aggregation, the plasmon of the aggregates would be brought in resonance with the excitation wavelength of 785 nm after 2 min. Finally, Xylenol Orange (XO) was chosen as the receptor. This ligand is a spectrophotometric indicator commonly used in the titration of heavy metal ions which displays two chelation sites having a strong affinity for Zn^2+^ at pH 7 (Figs. 1b and S2 and Tables S1 and S2)^36^. Moreover, XO potentially makes for a strong SERS reporter, as it displays itself a rich Raman spectrum thanks to its extended π system and multiple electron-rich centers.

### Peak assignments of SERS spectra

**Figure 2:**
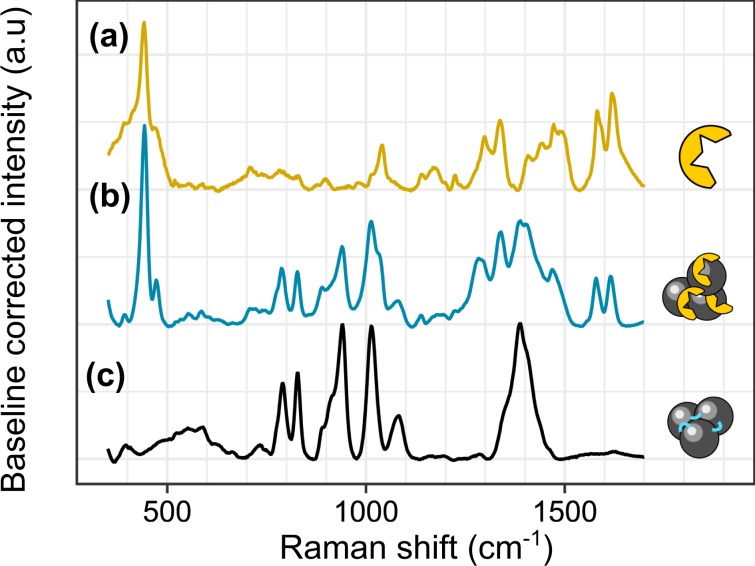
Normal Raman spectrum of Xylenol Orange (C_XO_ = 600 µM, 420 mW excitation power, 20 s acquisition) (**a**) and SERS spectra of spermine cross-linked aggregates of Ag NPs with (**b**) or without entrapped XO molecules (C_XO_ = 1.5 or 0 µM, 290 mW excitation, 7 s acquisition) (**c**).

The normal Raman spectrum of a dilute solution of XO (600 µM) and the SERS spectra of Ag NPs aggregated with spermine in the absence and in the presence of XO (1.5 µM) are presented in Fig. 2. Tentative peak assignments are summarised in Table S3. The most prominent features of the normal Raman spectrum of XO are a very strong peak at 442 cm^−1^ (out-of-plane wagging benzene mode), the sultone and sulfonate related stretching vibrations at 1039 cm^−1^, 1298 and 1338 cm^−1^, the multiple aromatic C = C stretching bands between 1400 and 1500 cm^−1^ and a doublet at 1579 and 1617 cm^−1^ typical within the xylenol family derived from C = C stretching modes of benzene^37–41^.

The SERS spectrum of Ag NPs cross-linked by spermine displays the characteristic vibrational features of citrates bound to the Ag surface, as reported by Munro *et al*.^42^, along with a doublet of peaks at 787 and 827 cm^−1^ that we assign to primary amine twisting and wagging modes^38^. The SERS spectrum of the XO-entrapping Ag NP aggregates displays a superimposition of peaks from citrate, spermine and XO, with the XO doublet about 1600 cm^−1^ and its 442 cm^−1^ peak sitting well outside of the fingerprints of spermine and citrate.

### SERS titration of XO bearing aggregates by Zn^2+^ ions

XO-loaded aggregates initially dispersed in pure water (blank sample) were titrated with increasing amounts of Zn^2+^ by spiking fifteen increasing volumes of a Zn standard into the into the dispersion. The sixteen spectra corresponding to the blank and Zn-supplemented samples are hereafter referred to as a titration series. Ten replicate titrations were conducted. The concentration of the Zn standard solution was independently analysed by ICP-OES. Over the titrations, XO concentration in the sample was maintained between 1.54 and 1.43 µM and Zn concentrations evenly spanned the range from 0.0 to 2.23 µM.

**Figure 3:**
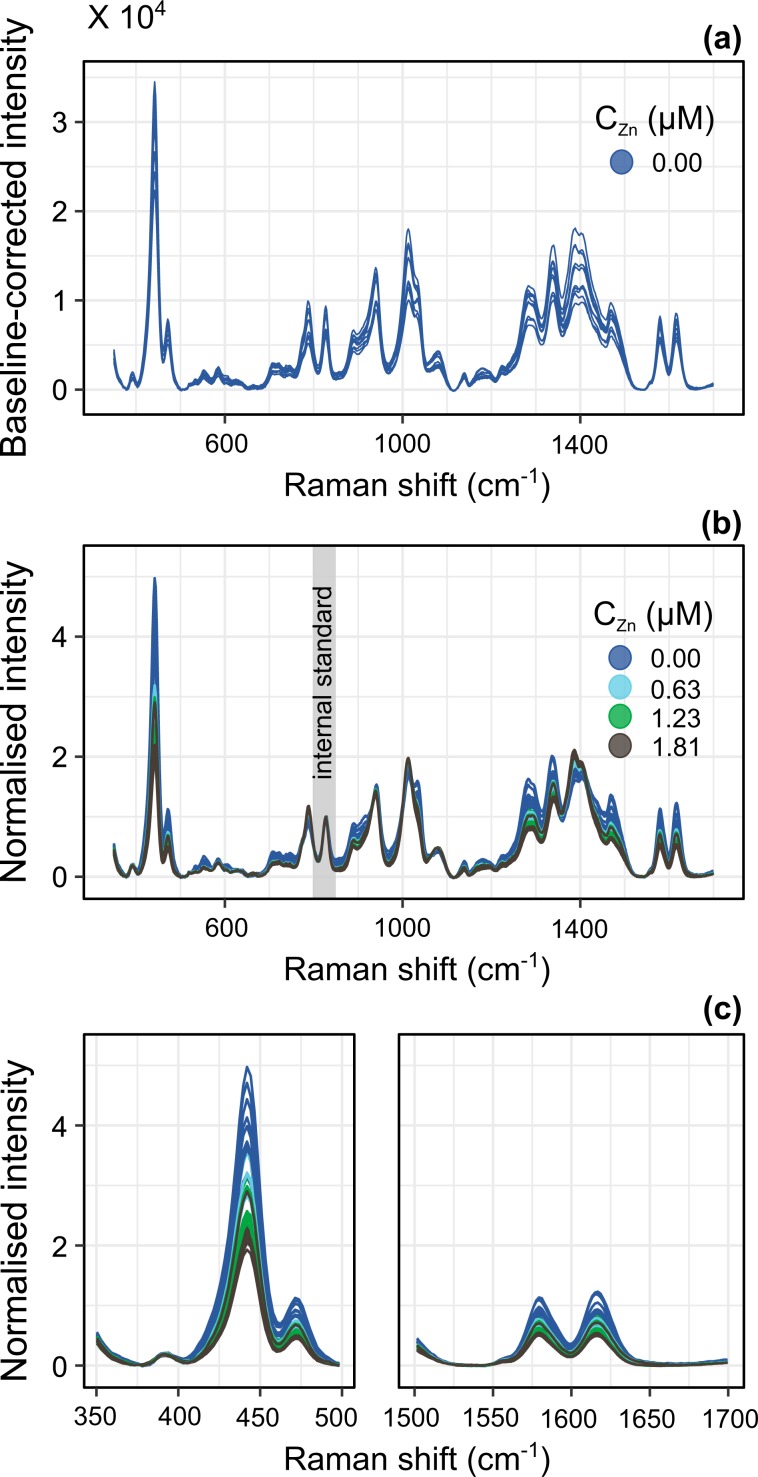
Baseline-corrected spectra acquired without added Zn^2+^ (10 replicates) (**a**); normalised spectra acquired upon increasing Zn^2+^ concentrations. Note that for clarity, only four concentrations out of every titration series are represented (40 out of the 160 recorded spectra). The grey area highlights the spermine peak used for normalisation (**b**); magnified views of the prominent peaks of XO (**c**).

As expected from the coarse chemical design, control over the aggregation process is poor, which results in irreproducible SERS count numbers in XO-loaded aggregates. Out of 10 baseline-corrected spectra acquired from replicate blank samples^43^, the intensities of the 941 cm^−1^ citrate peak and the 827 cm^−1^ spermine peak display relative standard deviations of 18% which indicate that the number of aggregates and/or their geometrical parameters are irreproducible (Fig. 3a). In an attempt to normalise the SERS spectra on the basis of the mean SERS enhancement factor for each sample, one of the spermine NH_2_ wagging and twisting peaks was used as an internal standard. To this end, all SERS spectra were subsequently normalised to the 827 cm^−1^ peak after baseline correction (Fig. 3b; normalisation to the 941 cm^−1^ peak gave similar results), according to Eq. (1).

1 $$I{(\bar{\nu })}_{norm}=I{(\bar{\nu })}_{baselinecorrected}/I{(\bar{\nu }=827)}_{baselinecorrected}$$

Over the titration, the intensities of all the peaks stemming from XO, and in particular those of the well-resolved peaks at 442, 1579 and 1617 cm^−1^, show a general decreasing trend, with no detectable shift in peak position (Fig. 3c). Speciation simulation shows that over the range of investigated Zn concentrations and at a pH of about 7, XO is quantitatively bound to Zn^2+^ ions (Fig. S3). However, as the SERS spectrum of XO does not feature any carboxylate-related vibration mode and as its phenol C-O stretching mode is likely buried into the intense C-O stretching mode of the alcohol group of citrate, the filling of the chelating sites of XO cannot be monitored directly. However, the chelation of Zn most likely comes with an important conformational change that would change the orientation of the XO molecules with regard to the SERS hot spots and hence modify their scattering cross-section. We hypothesise that this reorganisation of the receptor molecules upon complexation accounts for the observed drops in XO peak intensity. In the following, we examine the performance of the XO peak most affected by Zn recognition, namely that at 442 cm^−1^, as a predictor of the spiked Zn concentrations.

### Univariate calibration models

**Figure 4:**
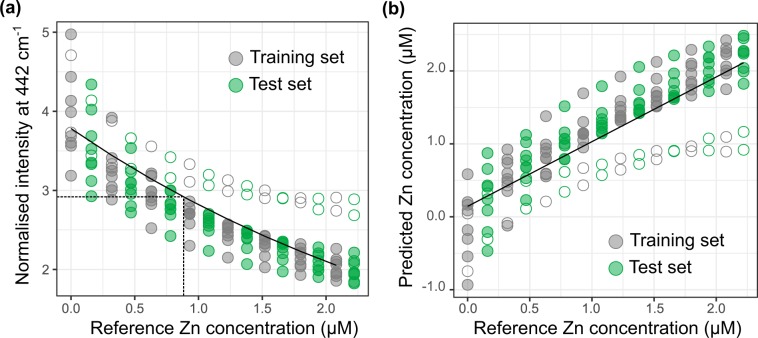
Evolution of the XO-specific 442 cm^−1^ peak (normalized to spermine 827 cm^−1^ peak) as a function of added Zn^2+^ concentration (**a**). The plain black line corresponds to the best exponential model fitting the training set (model **U1**); the dashed lines indicate the limit of detection. Empty circles highlight data points likely to be outliers. ${C}_{predicted}\,vs.{C}_{reference}$ validation plot of model **U1** (**b**). The plain black line corresponds to the linear fit of the predicted vs. reference concentrations (${\hat{C}}_{predicted}=a+b{C}_{reference}$).

Figure 4 **left** displays the evolution of the 442 cm^−1^ peak intensity as a function of Zn reference concentration for the 160 recorded spectra. The decrease in intensity with increasing concentration is monotonous. Despite the normalisation to the 827 cm^−1^ spermine peak, there is a 12% relative standard deviation (RSD) of the normalised intensity at each concentration within the 10 replicate titrations.

The dataset was divided in two equal parts for building (training set) and testing (test set) a calibration model, respectively. The training set is best fitted to an exponential model (model **U1;** Akaike’s information criterion was used to examine several dependencies^44^). With the usual definition for the limit of detection (LOD) as the concentration giving rise to a signal distant from that of the blank by three standard deviations of the blank^45^, the LOD was estimated at 883 nM. Over the course of the titration, the ligand concentration is nearly constant at 1.5 µM. Within the range of investigated Zn concentrations, complexation is nearly quantitative and at 1760 nM Zn, 95% of the ligand molecules are bound to a Zn ion. Hence, the 883 nM LOD of model **U1** means that about 50% of the sensitivity range of the ligand is useless.

From the relationship between signal intensity and concentration, model-predicted concentrations were calculated for the training and test sets. Each spectrum is then associated with two Zn concentrations: the actual (reference) Zn concentration in the sample and the estimate inferred from the signal intensity at 442 cm^−1^ using the **U1** model. The so-called ${C}_{predicted}\,vs.\,{C}_{reference}$ validation plot (or response plot) then gives a graphical depiction of the quality of the model (Fig. 4b) From its characteristics, the performances of the sensor can be derived (Fig. S4). An ideal unbiased model with perfect trueness would give rise to a linear plot with a slope of 1 and an offset of 0. Here, the slope, also known as the recovery rate, is satisfactory at 89%^46^. However, the predictive power of the model also takes into account its precision, which can be inferred from the root mean square error of prediction (RMSEP, Eq. (1)). To be truly representative of the predictive power of the sensor in unknown solutions, the RMSEP is best calculated on the basis of data not used for the calibration^30^. Therefore in the following we will reason on the root mean square error of validation (RMSEV), which is the RMSEP estimated on the test set used for validation.

2 $$RMSEP=\sqrt{\frac{{\sum }_{i=1}^{N}{({C}_{predicted,i}-{\hat{C}}_{predicted,i})}^{2}}{N}}$$

where *N* is the number of data points, ${C}_{predicted,i}$ is the predicted concentration of spectrum *i* dataset according to the calibration model (*eg*. **U1**) and ${\hat{C}}_{predicted,i}$ is the predicted concentration according to the linear fit of the predicted vs. reference concentrations (${\hat{C}}_{predicted}=a+b{C}_{reference}$).

For model **U1**, the RMSEV is of 392 nM, which corresponds to 35% of the median of the concentration range. To improve the precision on the estimate of analyte concentration, it is usual to combine several replicate measurements. Here we define the precision as the standard error of the mean predicted values, namely $RMSEV/\sqrt{n}$, where *n* is the number of replicate measurements. The precision can also be expressed in % relatively to the median of the sensitivity range. The RMSEV of model **U1** then translates into a precision of 11% when performing 10 replicate measurements, which is more than twice that usually encountered in ICP-MS.

Within the ten replicates of titration, two series graphically appear set apart from the rest of the dataset and present larger than average residuals (Fig. S5). Examination of normality plots for the residuals of the **U1** calibration model indicate that these series are likely outliers (Fig. S6). Removal of those two series of measurements leads to a calibration model (**U2**) with an RMSEV of 237 nM and an improved estimated LOD of 575 nM (Figs. S7–S9). However, as these improved figures of merit result from the removal of 20% of the initial dataset, they are not satisfactory.

In spite of the use of a spermine peak as an internal standard, the count numbers of the XO peaks are still highly variable. This is because as the XO molecules are entrapped at random during the aggregation process, which itself is ill-controlled, the XO insertion rate within the aggregate is also irreproducible. Not knowing exactly the concentration of the probe molecule is a common problem in fluorescence imaging, which is often circumvented by using ratiometric probes, *ie*. probes that present spectral features whose ratio vary as a function of their speciation^47,48^. Here however, all the most prominent and well-resolved peaks of XO, at 442, 1579 and 1617 cm^−1^, vary at the exact same rate during titration, in line with the assumption that the main driver of signal modification is the change of orientation of the molecule with regard to the Ag surface (Fig. S10). This precludes building a ratiometric calibration based on those well-defined peaks. However, in areas were the peaks of XO are superimposed onto those of citrate, such as the 900–950 cm^−1^, 1000–1100 cm^−1^ and 1250–1500 cm^−1^ intervals, more complex patterns of intensity variations arise as a function of Zn concentration. This indicates that taking into account more spectral data points might lead to a more fruitful ratiometric analysis.

### Multivariate calibration models

**Figure 5:**
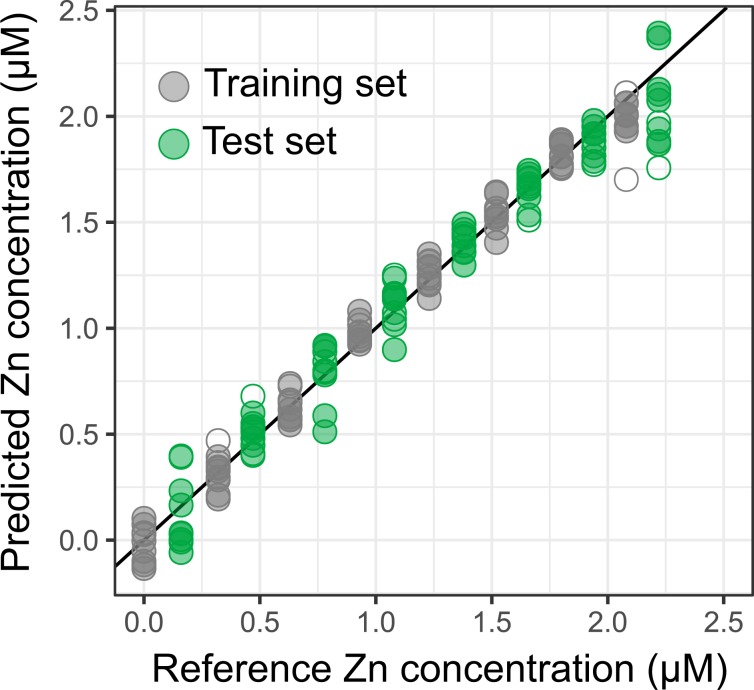
${C}_{predicted}\,vs.\,{C}_{reference}$ validation plot of PLS model **M50**. Empty symbols highlight data points previously identified as outliers in model **U1**.

Partial Least Square (PLS) regression is a multivariate calibration method especially designed to identify the relationships between data points that have the strongest correlation with analyte concentration^49^. A PLS regression model (**model M50**) was hence built and tested on the training and test datasets defined previously, after baseline correction by Satvisky-Golay filtering^50,51^. A leave-one-out cross-validation procedure was used to estimate the optimal number of components to include in the model and 10 components were finally retained (Fig. S11)^45^. The corresponding ${C}_{predicted}\,vs.{C}_{reference}$ validation plot displays an almost ideal slope of 96% and an RMSEV of 138 nM across the test set, which corresponds to a relative error of prediction of 12% relatively to the mean concentration of the investigated concentration range (Fig. 5). Upon acquisition of 10 replicate measurements, such error of prediction amounts to a precision of 4%. Noticeably, data points previously identified as outliers do not appear to stand out of the whole dataset. The distribution of the residuals of the best linear fit for the ${C}_{predicted}\,vs.{C}_{reference}$ plot appears also closer to normality than for the residuals of the previous univariate models, as evidenced by the corresponding normality plots (Figs. S12 and S13).

To the best of our knowledge, there are no definitive guidelines on the definition of the LOD in the case of multivariate regression. The usual definition of LOD in univariate regression that was used above implies a 7% probability of reporting a false positive or a false negative when reading a signal corresponding to the LOD^45^. By analogy with this usual definition, we defined our own LOD as the lowest possible concentration for which the chance of reporting a false positive or a false negative is less than 7%. This concentration corresponds to the lowest one that is statistically distinct from the blank sample at the 93% confidence level.

Practically, we serially examined whether the readouts for each of the 8 reference concentrations in the test set were significantly higher than the readouts of the blank sample in the training set. The LOD was determined as the lowest reference concentration for which the readout was significantly higher than that of the blank.

To this end, the residuals of the best linear fit for the ${C}_{predicted}\,vs.{C}_{reference}$ plot were assumed to be perfectly normally distributed, an assumption that is supported by the corresponding normality plots (Fig. S13) and a series of one-sided t-tests was performed between the predicted concentrations for the C_Zn_ = 0.00 µM samples and for the samples having increasing C_Zn_ concentrations (see Fig. S14 for a graphical description of the method). Note that the LOD determined in this way is actually an overestimation of the true one since the various concentrations in the test set are separated by 310 nM. The LOD was estimated at 160 nM (*P* = 0.01), which is the lowest concentration of the test set. Critically, this LOD is six times smaller than that of the univariate calibration **U1** model and four times smaller than the **U2** model, for which 20% of the data were discarded from the training and test sets as outliers, therefore evidencing that the PLS **M50** model vastly outperforms the univariate ones.

Using the same procedure, we estimated the limit of quantification (LOQ) of the **M50** model, this time setting the probability of reporting a false negative or false positive at 3.10^−5^% to match the usual definition of LOQ as the concentration giving a signal 10 standard deviations of the blank away from the signal of the blank itself. The LOQ was determined in this way to be 470 nM (*P* = 2 × 10^−11^), still below the LOD of the **U1** univariate calibration model.

Given the variability of the SERS signals, it is to be expected that the quality of the calibration model will depend on how much of this variability is included into the training set. To investigate this matter further, a series of additional calibration models was built in which the training set amounted to 40% (models **M40**) or 30% (models **M30**) of the data set while the test set remained the same as for model **M50**. Practically, this came down to discarding some titration series from the training set of model **M50**. Ten random draws of the discarded series were performed for each number of series to discard and the analytical performances were averaged over those draws (Table 1).

**Table 1:** Summary of analytical performances of investigated calibration models.

Name	Type	Training set	Testing set	Recovery (%)	RMSEV (nM)	Average precision (%)	LOD (nM)	LOQ (nM)
U1	Univariate	10 titration series	10 titration series	89	397	11%	883	—
U2	Univariate	8 titration series	8 titration series	88	237	7%	575	—
M50	Multivariate (PLS)	10 titration series	10 titration series	96	138	4%	160	470
M40	Multivariate (PLS)	8 titration series	10 titration series	97	162	5%	253	532
M30	Multivariate (PLS)	6 titration series	10 titration series	98	246	7%	346	898

Each titration series contains 16 spectra corresponding to 16 increasing *C*_*j*_ concentrations of Zn. For each model, the training and testing sets were built on data points with odd and even values of the concentration index *j*, respectively. The recovery rates, RMSEP, LOD and LOQ are determined from the ${C}_{predicted}\,vs.{C}_{reference}$ validation plots of each model. The average precision is estimated over 10 replicate measurements.

The recovery rate stays nearly constant and satisfactory over the whole range of sizes of training set. However, as expected the RMSEV increases with decreasing training set size. Consequently, The LOD and LOQ increase as well, highlighting the need for including many replicates of spectra when building the calibration model. Training sets accounting for 40% of the full data set provide the widest sensitivity range (250–2230 nM) for the smallest experimental calibration effort (64 spectral acquisitions).

## Discussion

Our sensor, with its LOD on the order of 200 nM for the best calibration models (M50 and M40), displays a sensitivity much weaker than what the SERS community has generally grown to expect. This sensitivity range stems in part from the frugality of the set-up: short sample preparation time makes for a less probable interaction between the SERS active areas and the analyte, while uncontrolled aggregation most likely increases the dispersion of the signal.

More important however is whether the proposed SERS sensing scheme bears any relevance to Zn monitoring in freshwater. Models **M40** and **M50** provide recovery rates (trueness) of about 97% and standard errors of the mean predicted values (precision) of about 4% relatively to the median of the investigated concentration range (over 10 replicate measurements). According to these metrics, our sensor compares favourably in terms of trueness and precision with AAS and ICP-OES, which are the most widely used methods for the determination of zinc in water samples. However, AAS and ICP-OES have LODs for Zn on the order of 10–15 nM, which are one order of magnitude smaller than the LOD of the **M50** model^52^. Still, not every monitoring context requires such sensitivity but many would benefit from a much higher measurement throughput and this is where the proposed SERS sensor would appear competitive.

According to the World Health Organisation, natural ground waters and surface waters have zinc concentrations between 150 and 610 nM and below 150 nM, respectively^53^. However, in former mining districts, zinc levels of stream water were shown to fluctuate between 300 nM and 1.5 µM over 24 h cycles due the circadian rhythm of photosynthetic activity of bacteria and algae^54^. In all of these contexts, some Zn concentrations might fell below the LOD or LOQ of the proposed SERS sensor. Yet it would have interesting value as a cheap way to gather large sets of data, to recognize trends and to flag abnormal Zn concentrations, in particular with regards to local environmental guideline values^55^, or to the sensitivity of endemic species to Zn (acute toxicity on some algae and fish species has been elicited from 460 and 1400 nM, respectively)^56^. Moreover, Cd levels in freshwaters strongly correlate with Zn levels. In Rhine and Seine river samples, two rivers impacted by intense human activities, mass concentration ratios of Cd to Zn on the order of 0.5% were reported^8,57^. In these samples, a measured Zn concentration of 1.5 µM would be indicative of a Cd concentration of 4 nM, which is above the freshwater guidelines values of 0.6 and 0.2 nM for Germany and France^58^. With its 160–2230 nM sensitivity range for Zn (using model **M50**), our sensor could therefore provide concentration estimations for the more deleterious element Cd without the need for ICP-OES.

Continuous or extensive monitoring of metal levels is a challenging problem due to the sheer volume of measurements needed to obtain meaningful time series or geographical overviews. The required mass budget is a limiting issue. Overall, we estimated that our frugal SERS sensor provides Zn readings for about 8.9 € per analysis, for the best performances, chemicals, consumables and labour included (see ESI for details of this estimation) using an instrument worth 20 k€. This amounts to 3–5 times less than what is usually charged for a metal analysis in a public or private sector analytical laboratory that uses an instrument worth at least twice as much as our portable Raman spectrometer. Moreover, the whole set-up of the proposed SERS sensing scheme does not require the complicated or hazardous lab supplies (gas cylinder or concentrated acids) needed in AAS or ICP-OES which impose tedious safety constraints. In addition, the portable spectrometer used in this study is truly compact (3.5 kg). Therefore the whole set-up could easily be brought to the water point to provide readings on the spot. We believe that the proposed sensing set-up could therefore open the way to a more democratised monitoring of freshwater quality.

## Conclusion

SERS usually stands as a pricey and impractical technique because of the cost of SERS-active materials or of labour-intensive sample preparation technique. In this work, we examined the possibility of performing SERS detection frugally on the case of the freshwater contaminant Zn^2+^. The chosen chemical components can be easily sourced on a scale worth 5,000 measurements, an affordable portable spectrometer was used and sample preparation and spectral acquisition amounted to a couple of minutes. The large dataset acquired enabled an in-depth investigation of the analytical performances.

Due to the simple chemical design, SERS count numbers were found to be highly variable, which precluded the use of univariate calibration models to quantify Zn^2+^. However, calibration models built on the multivariate PLS algorithm proved much better at dealing with this variability and displayed superior analytical performances. The trueness and precisions of predictions were comparable to those of ICP-OES or AAS. However, the limits of detection were found to be one order of magnitude higher. Despite this, our frugal SERS sensor was sensitive at concentrations relevant for freshwater water analysis. In addition and in contrast to those golden standard techniques, it could easily be implemented on the field while giving readings for a fraction of their respective costs per analysis. Therefore, we suggest that the proposed sensor could be used in complement to ICP-OES and AAS either to flag alarming Zn concentrations, possibly as a proxy for more deleterious and less abundant heavy metals, or as a cheap way to monitor very large assemblies of samples. However, before it can make its way into field monitoring campaign, there needs to be some demonstration of its validity in natural water samples. Freshwaters contain suspended colloids, dissolved organic matter, electrolytes and competing cations which could interfere with the detection. Moreover, the concentrations of these potential interfering species vary to a large extent depending on the weather events and seasons. Validating a sensor in these complex, fluctuating mixtures will require a vast set of conditions to be explored. Only a frugal design can afford it.

## Methods

### Chemicals

Silver nitrate (≥99.99%, Alfa Aesar), xylenol orange disodium salt (Aldrich), trisodium citrate dihydrate (≥99.0%, Aldrich), spermine tetrahydrochloride (99%, Alfa Aesar), zinc nitrate hexahydrate (≥99.0, Aldrich), nitric acid (69%, AnalR Normapur), sodium hydroxide 1 M solution (Fluka) were used as received from the suppliers. Dilutions were performed using ultrapure water fractions having a resistivity of 18 MΩ.cm when exiting the purification apparatus and kept for no longer than 1 day.

### Synthesis of Ag nanoparticles (NPs)

100 mg silver nitrate were dissolved in 500 mL ultrapure water in an Erlenmeyer flask cleaned with aqua regia immediately prior to the synthesis. The silver solution was brought to the boil. Next, 10 mL of a 1% wt trisodium citrate dihydrate solution were quickly added under vigorous stirring. Within 5 min, the reaction mixture turned pale yellow then the colour intensified to finally lead to a milky green solution. The solution was left to stir at 90 °C for 1 hour and then stored shielded from light at 4 °C. The NPs need to age under these conditions for a couple of days before being put to use in the proposed sensing scheme (in order to always have NPs available for SERS measurements, a batch can be prepared every other day). The SERS data presented here were collected at day 5 after synthesis. Within the frame of the proposed sensing scheme, we acquired satisfactory spectra using NPs that had aged from 5 to 20 days. In addition, we found that these NPs to be stable and functional for SERS experiments for a couple months after their synthesis when stored at 4 °C shielded from light (Figs. S16 and S17).

### Stock solutions for analysis

Stock solutions of Zn^2+^, xylenol orange and spermine were prepared from zinc nitrate hexahydrate, xylenol orange disodium salt and spermine tetrahydrochloride respectively. Each solution was adjusted to pH 7.0 using 0.1 M sodium hydroxide and 0.1 M nitric acid solutions. The XO stock solution had a target concentration of 30.7 µM, the spermine stock solution had a target concentration of 149 µM and the zinc solution had a concentration of 31.9 µM as determined by ICP-OES.

### Spectral acquisition and sample preparation

The spectra were acquired on a BWTEK iRaman Plus portable spectrometer equipped with a 785 nm laser having a maximal power of 420 mW. Excitation and collection were performed in backscattering configuration using a coaxial optical fibre Raman probe. The spectrometer resolution is ~4.5 cm^−1^. The spectral acquisition range was set to 300–2000 cm^−1^. The acquisition time was set to 7 s and the laser power to 70%. Each spectrum was averaged over 3 spectral recordings. A titration series consists of 16 spectra acquired as follows. In a 4.5 mL spectrophotometry quartz cuvette, 100 µL of NP stock solution were mixed with 100 µL of XO stock solution using a vortex mixer. Next 10 µL of spermine solution were added and the mixture was rapidly homogenised. Then 1790 µL of ultrapure water having a pH of 7 were added in the cuvette and the mixture further homogenised to give rise to a Zn-free standard sample of which a spectrum was acquired exactly 2 min after the addition of spermine. Subsequently, 10 µL of Zn standard solution were spiked into the cuvette and the mixture was homogenised with a vortex mixer before acquiring its spectrum. In total over a titration series, 15 aliquots of 10 µL of Zn standard solution were mixed in the cuvette to achieve an upper Zn concentration of  2.23 µM. 10 titrations series were conducted.

### Data analysis

Spectral data were processed using the open-source R software^59^. Baseline corrections were performed with the modpolyfit() function (polynomial fitting^43^) of the *baseline* package for univariate analysis and the sg() function (Satvitsky-Golay baseline correction^50^) of the *signal* package for multivariate analysis^60,61^. Partial Least Squares regression was performed using the *pls* package. Speciation analysis was conducted using Visual MINTEQ software with the speciation constants given in Tables S1 and S2 ^36,62,63^.

## Supplementary information

Supporting Information.

Dataset.
