# Supplementary material for: A frugal implementation of Surface Enhanced Raman Scattering for sensing Zn2+ in freshwaters – In depth investigation of the analytical performances
Source: Sci Rep. 2020 Feb 5;10:1883. doi: 10.1038/s41598-020-58647-7 (PMC7002737; doi:10.1038/s41598-020-58647-7)
Supplement: Supplementary file 1 — Supporting Information. [file 41598_2020_58647_MOESM1_ESM.pdf]

A frugal implementation of Surface Enhanced Raman Scattering for sensing Zn^{2+} in freshwaters – in depth investigation of the analytical performances.

SUPPORTING INFORMATION

Gwennhaël Brackx,[a] Damien Guinoiseau,[b] Ludovic Duponchel,[c] Alexandre Gélabert,[b] Victoria Reichel,[a] Samia Zrig,[d] Jean-Marc Di Meglio,[a] Marc F. Benedetti,[b] Jérôme Gaillardet[b] and Gaëlle Charron.[a,*]

[a] Laboratoire Matière et Systèmes Complexes, UMR 7057, Université Paris Diderot, Sorbonne Paris Cité, CNRS, 10 rue Alice Domon et Léonie Duquet, 75205 Paris cedex 13, France.

[b] Institut de Physique du Globe de Paris, Sorbonne Paris Cité, CNRS UMR 7154, 1 rue Jussieu, 75005 Paris, France

[c] LASIR CNRS UMR 8516, Université de Lille, Sciences et Technologies, 59655 Villeneuve d'Ascq Cedex, France.

[d] ITODYS, UMR 7086, Université Paris Diderot, Sorbonne Paris Cité, CNRS, 15 rue J-A de Baïf,, 75205 Paris cedex 13, France

1 Transduction and recognition properties of the sensor

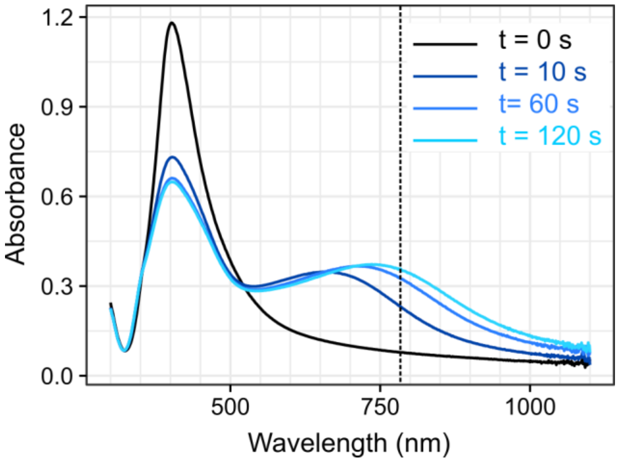

Figure S1: Absorption spectra of Lee-Meisel Ag NPs upon interaction with spermine as a function of time. The spectra were obtained by mixing in a quartz cuvette 100 µL of as synthesised NPs with 10 µL of a 0.15 mM spermine solution and subsequently adjusting the total volume to 2 mL. The stock solutions were adjusted to a pH of 7 prior to mixing. The dashed line indicate the wavelength of the laser used for Raman acquistions.

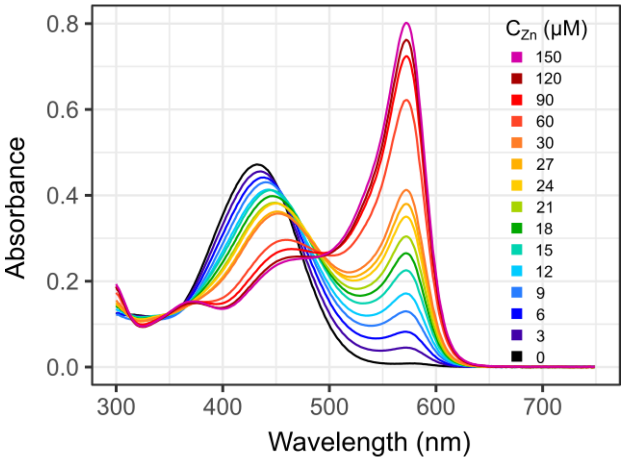

Figure S2: Absorption spectra of a 24 μM aqueous solution of XO at pH 7 upon titration with Zn^{2+}. The legend refers to the final analytical concentration of Zn in the mixture.

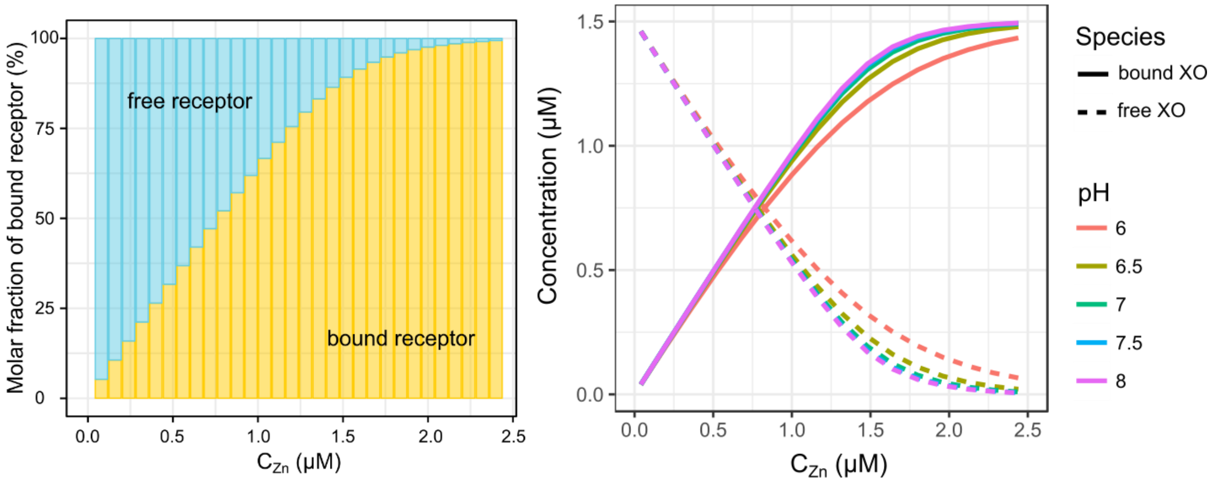

Figure S3: Evolution of molar fraction of bound receptor as a function of Zn concentration (*left*). Note that at a pH of 7 and within the investigated Zn concentration range, the free ligand exists in two main forms (three times and four times deprotonated respectively) and the bound ligand exists in two forms with one or two chelated Zn^{2+} ions respectively. Here the free receptor and bound receptor molar fractions represent the sum of the free forms and bound forms respectively. The speciation is largely unaffected by pH fluctuations about 7 (*right*).

Useful constants for speciation analysis:

$$iZn^{2+} + jH^{+} + kL^{6-} \rightleftarrows \left[Zn_iH_jL_k\right]^{-6+2i+j}$$

$$\beta_{ijk} = \frac{\left[Zn_iH_jL_k\right]}{[Zn]^i[H]^j[L]^k}$$

logβ_{101}	15.41 ± 0.06
logβ_{111}	24.91 ± 0.05
logβ_{121}	29.84 ± 0.04
logβ_{201}	25.37 ± 0.03

Table S1: Formation constants for the zinc-xylenol system.[1]

$$H_{n+1-q}XO + H_2O \rightleftarrows H_{n-q}XO^{-q} + H_3O^{+}$$

$$K_{a,q} = \frac{\left[H_{n-q}XO^{-q}\right][H_3O^{+}]}{\left[H_{n+1-q}XO\right]}$$

$-logK_{a,1}$	1.5
$-logK_{a,2}$	2.32 ± 0.15
$-logK_{a,3}$	2.85 ± 0.06
$-logK_{a,4}$	6.67 ± 0.03
$-logK_{a,5}$	10.39 ± 0.05
$-logK_{a,6}$	12.23 ± 0.05

Table S2: Acidity constants of Xylenol Orange.[2,3]

SERS peak assignment:

Peak (cm^{-1})	Assignment	Ref.
442	$\omega_{ring, out-of-plane}$, XO	[4–7]
787 & 827	ω_{NH2} and τ_{NH2}, spermine	[4–8]
903, 935 & 950	ν_{C-COO-}, citrate	[9]
1023	ν_{C-O}, citrate	[9]
1039	$\nu_{S=O, symmetric}$, XO or $\omega_{ring, in-plane}$, XO	[4–7]
1092	$\nu_{C-N, primary}$, spermine	[4]
1298 & 1338	$\nu_{S=O, asymmetric}$, XO	[4,7,8]
1383	ν_{COO}, citrate	[9]
1400-1500, multiple features	$\nu_{C=C, ring}$, XO	[4,7,8]
1579 & 1617	ν_{8a} et ν_{8b} benzene-like	[4,7,8]

Table S3: List of observed Raman and SERS peaks and corresponding tentative assignments.

2 Chemometric analysis

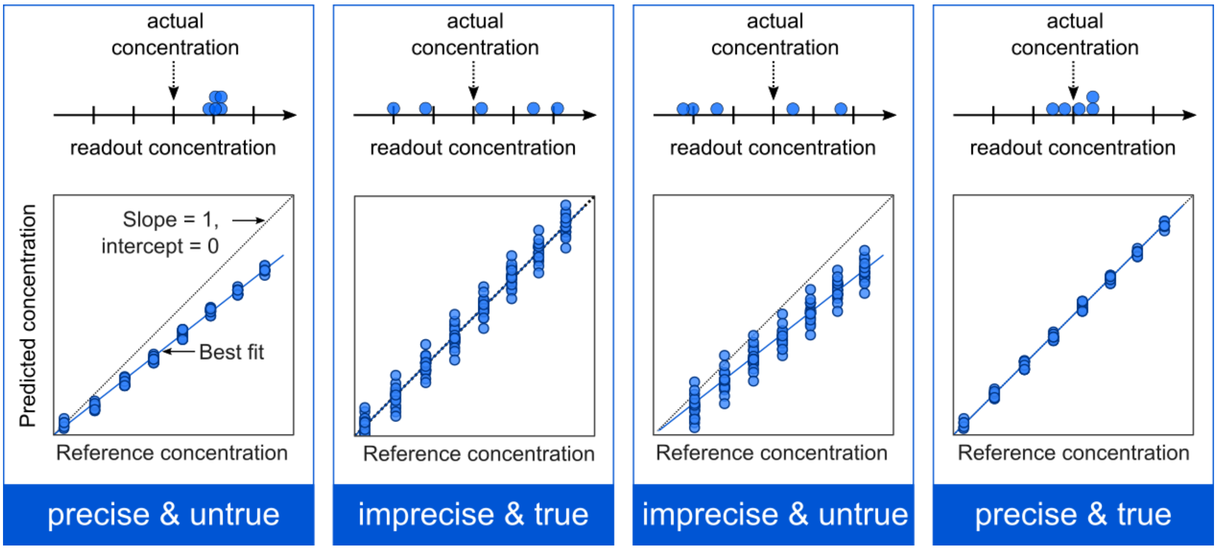

Figure S4: Schematics of information that can be derived from validation plots, also called response plots.

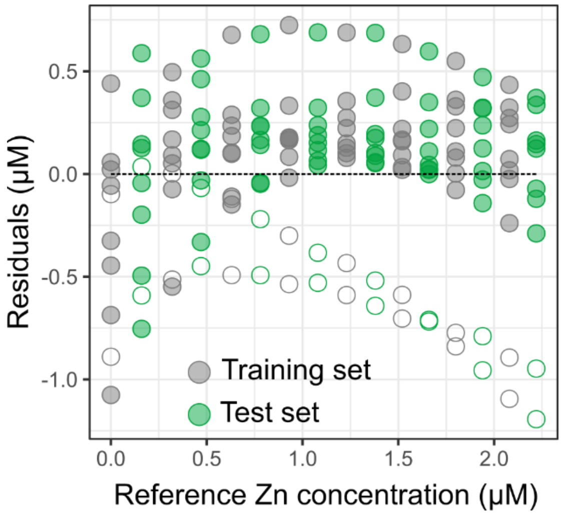

Figure S5: Distribution of residuals e_i ($e_i = C_{predicted,i} - \widehat{C}_{predicted,i}$) of model **U1** across the concentration range. Empty circles highlight data points likely to be outliers.

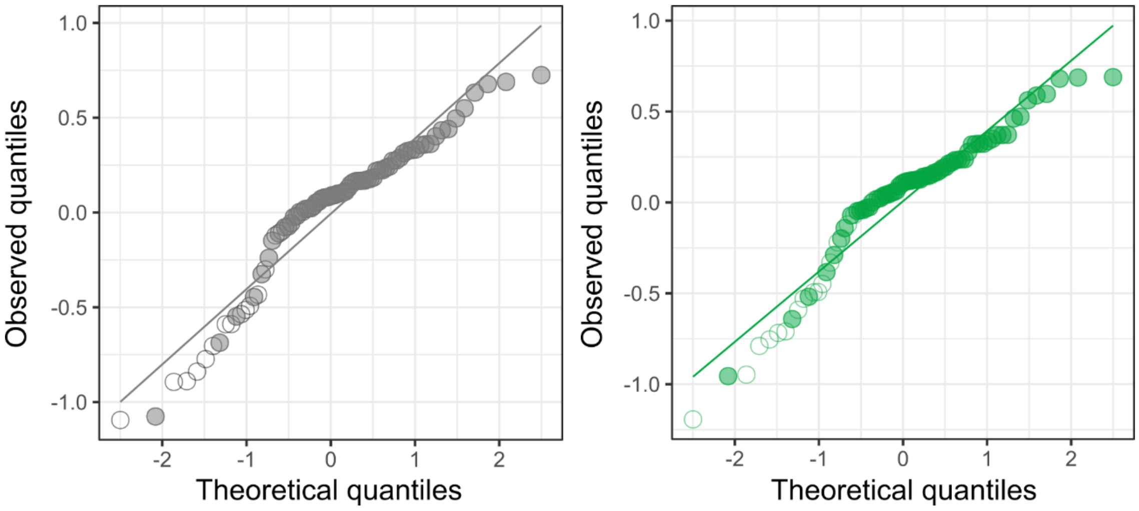

Figure S6: Normality plots of the residuals of the exponential **U1** model fitting the dependence of the 442 cm^{-1} peak normalised intensity to the reference Zn^{2+} concentration, for the training set (*left*) and the test set (*right*). Empty circles correspond to the data points suspected of being outliers.

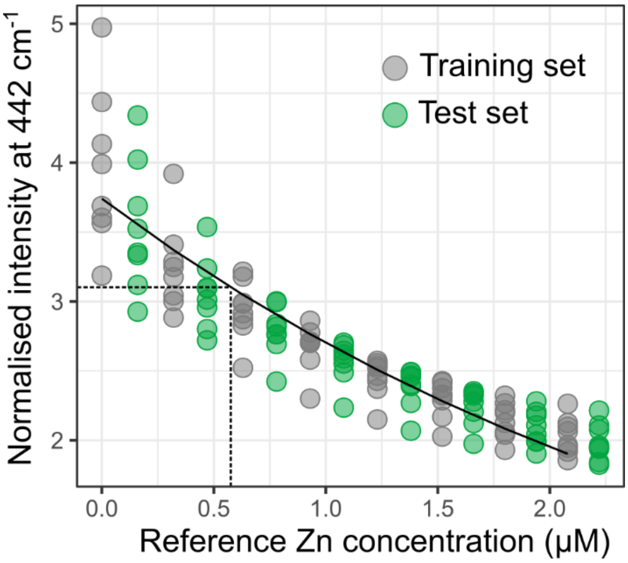

Figure S7: Evolution of the XO specific 442 cm^{-1} peak (normalized to spermine 827 cm^{-1} peak) as a function of added Zn^{2+} concentration within the dataset freed from suspected outlier series. The plain black line corresponds to the best exponential model fitting the training set (model **U2**); the dashed lines indicate the limit of detection.

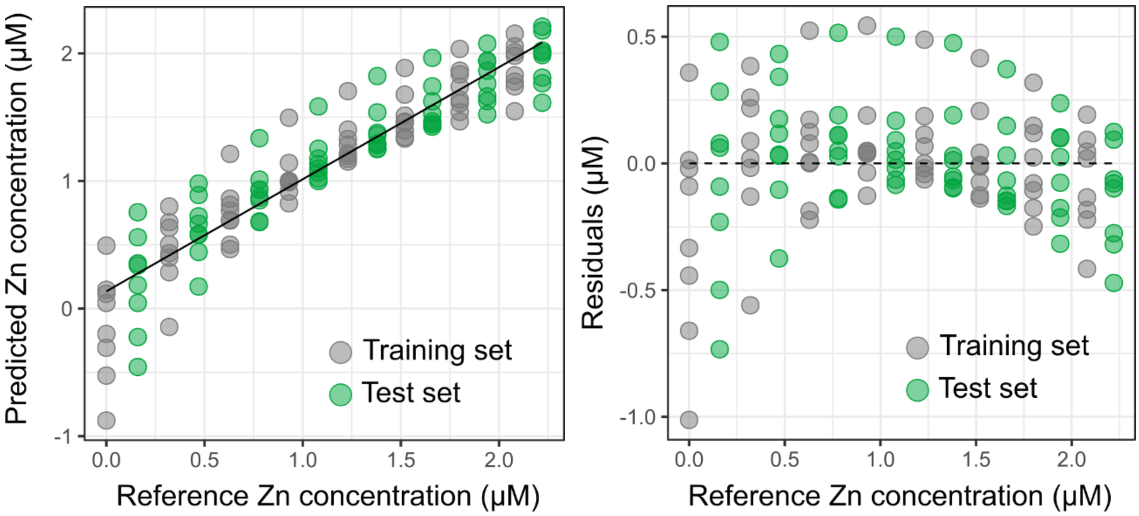

Figure S8: $C_{predicted}\ vs.\ C_{reference}$ validation plot of model **U2** (*left*). The plain black line corresponds to the linear fit of the predicted vs. reference concentration ($\widehat{C}_{predicted} = a + b\ C_{reference}$). Distribution of residuals e_i of model **U2** across the concentration range (*right*).

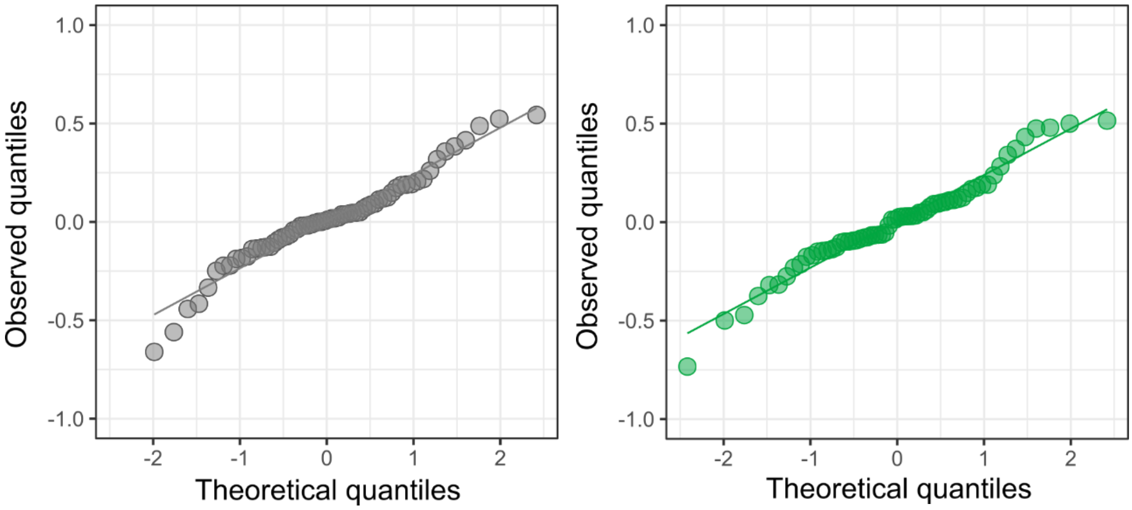

Figure S9: Normality plots of the residuals of the exponential **U2** model fitting the dependence of the 442 cm^{-1} peak normalised intensity to the reference Zn^{2+} concentration within the restricted dataset freed from suspected outliers, for the training set (*left*) and the test set (*right*).

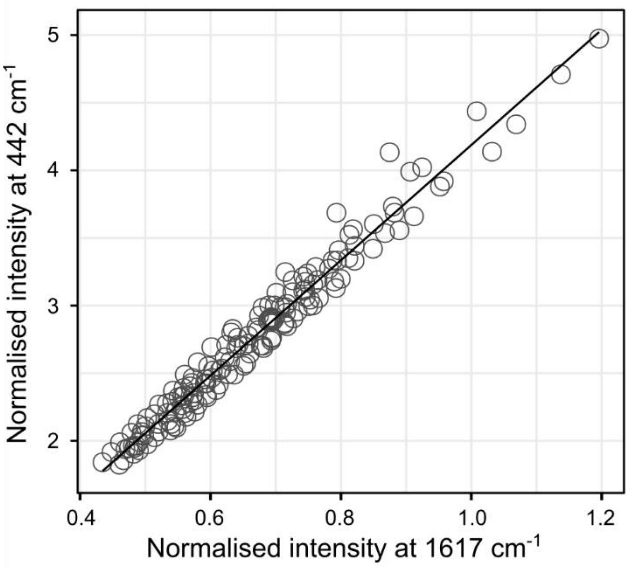

Figure S10: Evolution of the XO specific 442 cm^{-1} peak as a function of the XO specific 1617 cm^{-1} peak (both peaks normalized to spermine 827 cm^{-1} peak).

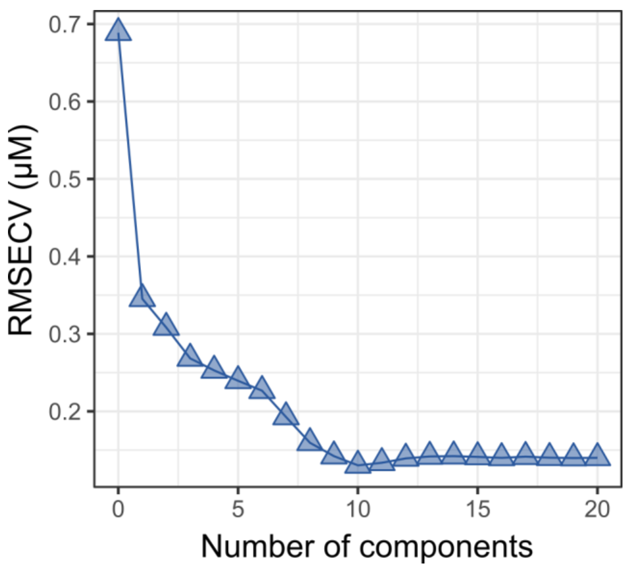

Figure S11: Evolution of the root mean square error of cross-validation (RMSECV, based on the leave-one-out procedure) as a function of components included in the PLS calibration model.

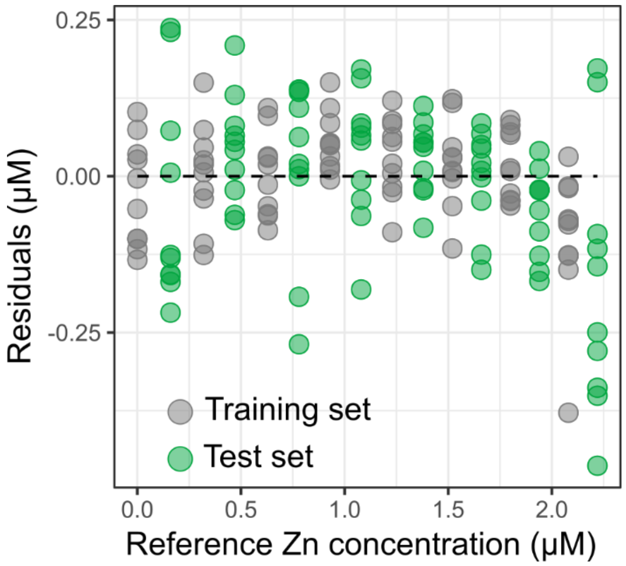

Figure S12: Residuals of the **M50** model.

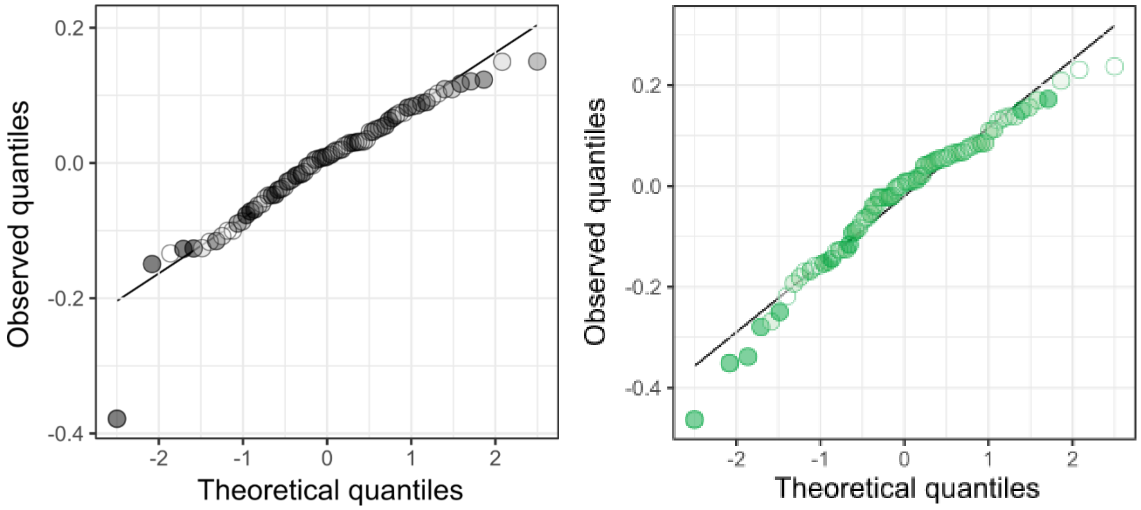

Figure S13: Normality plots of the residuals of the **M50** PLS model displayed in **Fig. 5**, for the training set (*left*) and the test set (*right*). The opacity of the symbols is mapped to the reference zinc concentration, with the darker symbols corresponding to the highest Zn concentration.

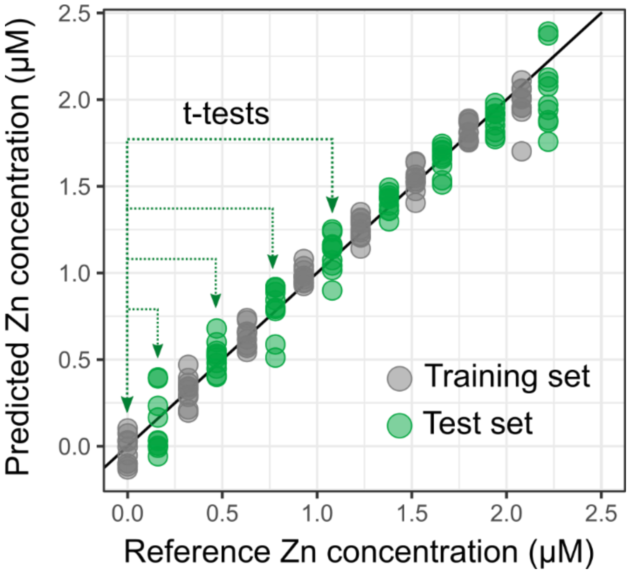

Figure S14: Graphical depiction of the data groups selected for the t-tests aimed at determining the LOD and LOQ.

3 Estimation of cost per analysis

The cost of performing one spectral acquisition has two main contributors: the consumption of stock solutions of NPs, spermine, XO and Zn standard, and the labour time needed to prepare the sample in the cuvette and record the corresponding spectrum. We estimate below the costs of these contributors.

1. **Consumption of stock solutions of NPs, spermine, XO and Zn standard:**

Consumption of chemicals from commercial supplier:

chemical	Silver NPs	XO	spermine	Zinc nitrate
Volume consumed	100 µL	100 µL	10 µL	75 µL on average
Chemicals	Silver nitrate 99.999% pure, trisodium citrate dihydrate	Xylenolorange di sodium salt	Spermine tetrahydrochloride	Zinc nitrate hexahydrate, >99.0%
Stock concentration	1.177 mM	30.7 µM	149 µM	31.9 µM
Cost per acquisition	275 µ€	71 µ€	13 µ€	0.04 µ€
Cost for 5000 acquisitions	1.375 €	0.355 €	0.065 €	0.2 m€

Labour for the preparation of the stock solutions:

The synthesis of the nanoparticles at a scale of 500 mL is completed in one hour, within which the setting-up of the reaction mixture only takes the first 10 min. During the remaining 50 min of NP growth, the stock solutions of spermine, xylenol orange and zinc nitrate can be prepared and adjusted in pH. Reasoning in terms of PhD labour cost, these operations amount to 18€/hour (charged cost).

Overall cost of consumption of stock solution:

The overall cost is dominated by the labour time needed to prepare the NPs and the XO, spermine and Zn solutions. Hence the consumption of stock solution is estimated to cost 18€ for 5000 acquisitions, or 4 m€ per acquisition. This cost is about 3 order of magnitude lower than the cost of spectral acquisition. It will therefore be hereafter neglected.

2. SERS acquisition:

For calibration:

First, a Zn-free standard sample is prepared by mixing the NP, XO and spermine solutions in the cuvette and then addding pure water and mixing again. The spectrum is then recorded. An aliquot of Zn concentrated standard is then spiked into the cuvette, which is further mixed and a new spectrum is recorded. The procedure is repeated to span the desired Zn sensitivity range (**Fig. S15**). The cuvette is then cleaned in an ultrasonic bath and another titration series is conducted. To build a training set equivalent to that used for building the M50 calibration model, 8 concentration steps are explored in each titration series and 10 replicate titrations must be conducted. From the performed measurements, we estimated that each titration series takes on average 9' to complete. Acquisition of the training set therefore takes 90 min which amounts to 27€ worth of labour.

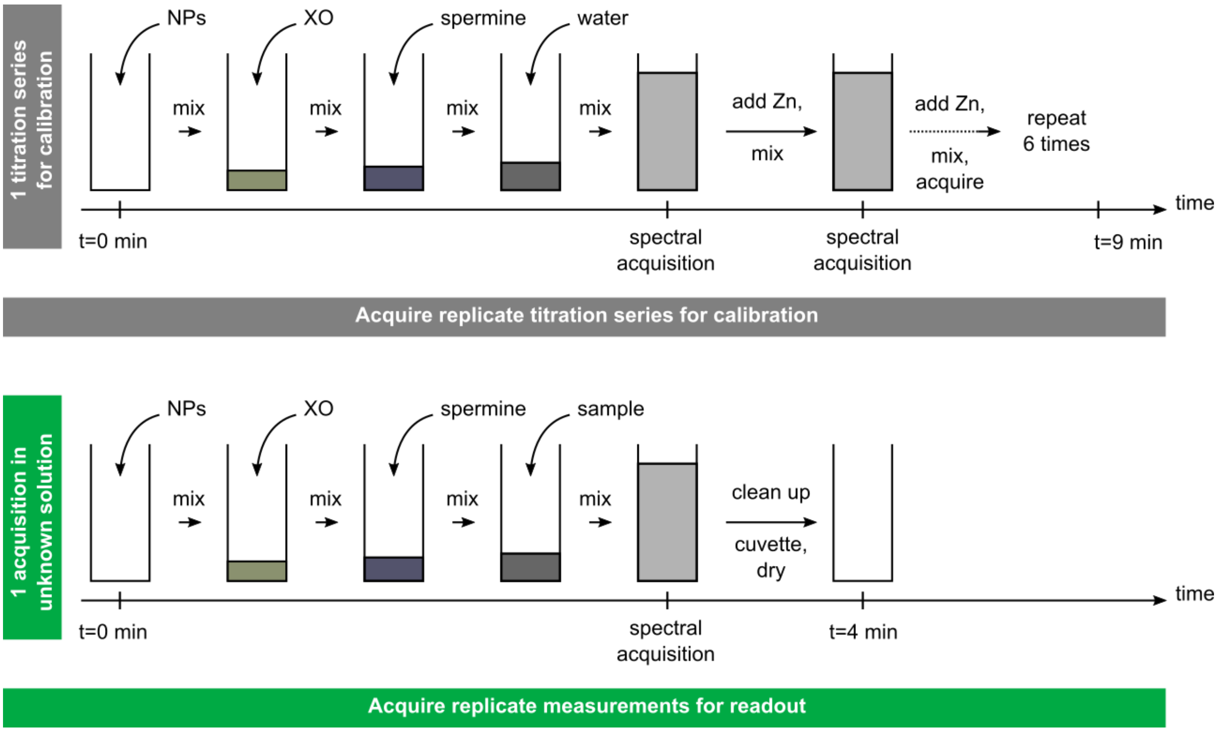

Figure S15 : graphical depiction of the timeline of sample preparation, for calibration or for acquisition in an unknown solution. Note that 10 titrations series are needed to achieve the analytical performances of model M50 and 6 acquisitions on the unknown solution are needed to give readouts with 5 % precision.

For Zn determinations on unknown samples:

The NP, XO and spermine solutions are mixed in the cuvette and then the water sample is added and the cuvette is again homogenised. The spectrum is then recorded and the cuvette is cleaned. The whole acquisition procedure can be comfortably performed in 4 min (**Fig. S15**), which amounts to 1.2€ worth of labour. To achieve a standard error of the mean predicted Zn concentrations of 5% at most, 6 replicate measurements are needed and therefore each mean determination of Zn concentration costs 7.2 € worth of labour.

3. **Cost per Zn determination, including calibration**

Assuming that the spectrometer is deployed in a neighbourhood for one day (8 hours), 6 hours 30 min can be dedicated to actual sample measurements after the 1h30 calibration, corresponding to 98 spectral acquisitions or 16 determinations of Zn concentration. The overall cost of each of these Zn readings, including calibration is therefore 8.9€.

4 Ageing and stability of Lee-Meisel NPs

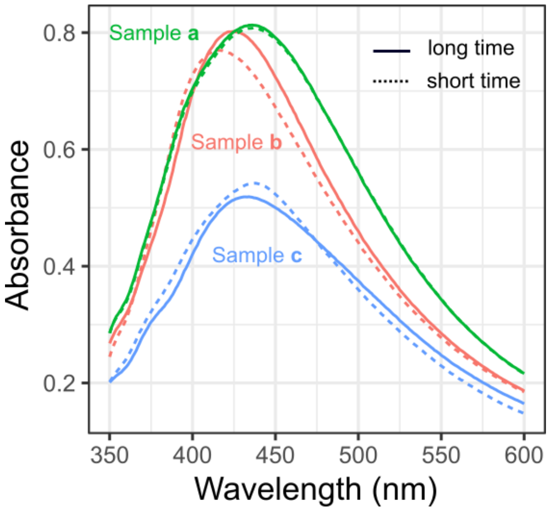

Figure S16: Extinction spectra of Lee-Meisel NPs (diluted 10 fold) at short times (day 1 for batches **a** & **b** and day 3 for batch **c**) and long times (day 108 for batches **a** & **b** and day 118 for batch **c**) after their synthesis. The NPs have been stored at 4°C shielded from light in the meantime.

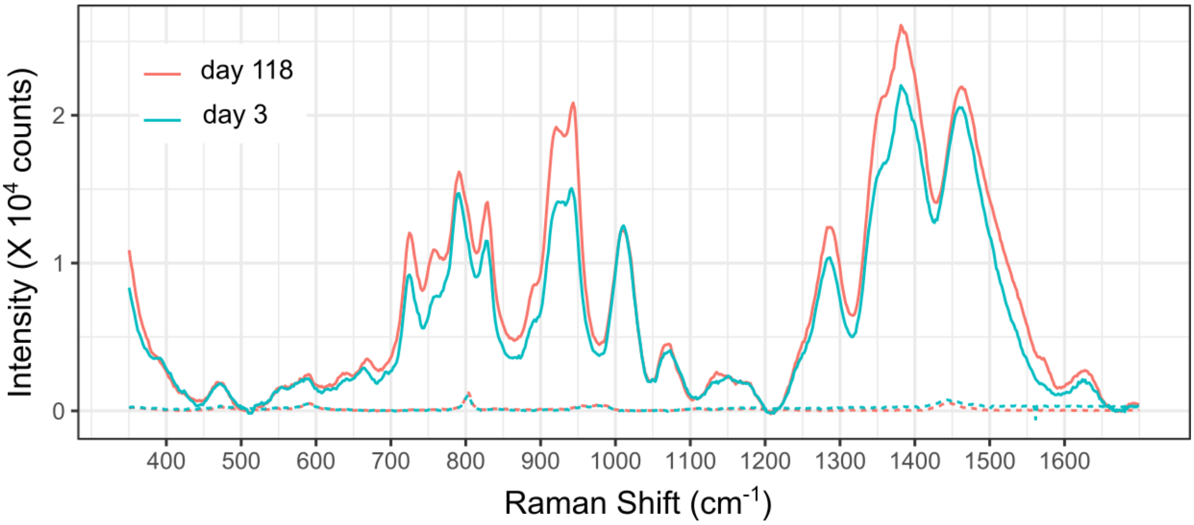

Figure S17: SERS spectra of Lee-Meisel NPs aggregated in the presence of spermine (C_{Ag} = 575 µM and $C_{spermine}$ = 7 µM) at day 3 and day 118 after their synthesis, evidencing comparable SERS intensity counts. The NP batch used is the **c** one in **Figure S16**. The dashed lines correspond to the normal Raman signals of the PMMA spectrophotometric cuvettes.

5 References

(1) Sato, H.; Yokoyama, Y.; Momoki, K. Purification of Xylenol Orange by Ion-Exchange Chromatography, and Chelate Formation with Lead(II) and Zinc(II). *Anal. Chim. Acta* **1977**, *94* (1), 217–220.

(2) Suzuki, T.; Tiwari, D.; Hioki, A. Precise Chelatometric Titrations of Zinc, Cadmium, and Lead with Molecular Spectroscopy. *Anal. Sci.* **2007**, *23* (10), 1215–1220.

(3) Murakami, M.; Yoshino, T.; Harasawa, S. Separation and Acid Equilibria of Xylenol Orange and Semi-Xylenol Orange. *Talanta* **1967**, *14* (11), 1293–1307.

(4) Socrates, G. *Infrared and Raman Characteristic Group Frequencies: Tables and Charts*, 3rd Edition.; Wiley-Blackwell: Chichester, 2004.

(5) Xylenol Blue sodium salt 114561 https://www.sigmaaldrich.com/catalog/product/aldrich/114561 (accessed May 11, 2018).

(6) Cresol Red sodium salt 114480 https://www.sigmaaldrich.com/catalog/product/aldrich/114480 (accessed May 11, 2018).

(7) Bromocresol Purple sodium salt 860891 https://www.sigmaaldrich.com/catalog/product/aldrich/860891 (accessed May 11, 2018).

(8) Mayo, D. W.; recherche, résultats de; Hannah, R. W. *Course Notes on the Interpretation of Infrared and Raman Spectra*, Re-issue.; Wiley-Blackwell: Hoboken, N.J, 2004.

(9) Munro, C. H.; Smith, W. E.; Garner, M.; Clarkson, J.; White, P. C. Characterization of the Surface of a Citrate-Reduced Colloid Optimized for Use as a Substrate for Surface-Enhanced Resonance Raman Scattering. *Langmuir* **1995**, *11* (10), 3712–3720.
